# Impaired emotion regulation in obsessive-compulsive disorder and hoarding disorder

**DOI:** 10.1192/j.eurpsy.2021.1119

**Published:** 2021-08-13

**Authors:** M. Puialto Amieiro

**Affiliations:** Psychiatry, Hospital Bellvitge, Barcelona, Spain

**Keywords:** Obesessive Compulsive Disorder, Hoarding Disorder, Emotional Regulation, Obsessiveness

## Abstract

**Introduction:**

There is suggestive evidence linking hoarding with several problems in emotional regulation, and though this is shared with OCD patients, it may not correlate to the presence of obsessive symptoms.

**Objectives:**

The present study aimed to examine self-reported deficits in emotion regulation (ER) and obsessiveness among individuals with hoarding disorder (HD) in comparison with others with obsessive compulsive disorder (OCD) and healthy controls

**Methods:**

Twenty-two adult outpatients with HD, twenty-two with OCD and twenty-two age and gender matched healthy control (HC) participants completed the Emotion Regulation Questionnaire (ERQ) which measures respondents tendency to regulate their emotions in two ways: Cognitive Reappraisal and Expressive Suppression. They fulfilled as well the OCI-R which evaluates six groups of OCD symptoms: Washing, Checking, Ordering, Obsessing, Hoarding, and Neutralizing.

**Results:**

The HD and OCD groups scored higher, (p 0.04), on Cognitive Reappraisal than did the HC group. There was no significant difference between groups in Expressive Suppression. HD and HC groups scored significantly lower, (p < 0.001), in OCI-R than OCD patients.

**Conclusions:**

Results suggest that OCD and HD are characterized by self-reported deficits in ER, but this relationship in HD patients is not solely attributable to obsessive symptoms.

